# Rapid and Economical Drug-Eluting IOL Preparation via Thermoresponsive Agarose Coating for Effective Posterior Capsular Opacification Prevention

**DOI:** 10.3389/fbioe.2022.930540

**Published:** 2022-08-05

**Authors:** Siqi Chen, Chen Qin, Qiuna Fang, Lan Duo, Mengting Wang, Zhennv Deng, Hao Chen, Quankui Lin

**Affiliations:** Department of Biomaterials, School of Ophthalmology and Optometry, Eye Hospital, Wenzhou Medical University, Wenzhou, China

**Keywords:** agarose, surface modification, drug-eluting coating, intraocular lens, posterior capsular opacification

## Abstract

Posterior capsular opacification (PCO), the highest incidence complication after cataract surgery, is mainly due to the attachment, proliferation, and migration of the residual lens epithelial cells (LECs). Although the drug-eluting IOLs have been proved to be an effective way to prevent PCO incidence, its preparations are time consuming and require tedious preparation steps. Herein, the thermoreversible agarose is adopted to prepare drug-eluting IOL. Such functional coating can be obtained easily by simple immersion in the antiproliferative drug containing hot agarose and taken out for cooling, which not only does not affect the optical property but also can effectively decrease the PCO incidence after intraocular implantation. As a result, the proposed agarose coating provides a rapid and economical alternative of drug-eluting IOL fabrication for PCO prevention.

## 1 Introduction

Cataract, the opacification of the lens caused by various reasons, is the most common blinding eye disease in the world (Khairallah et al., 2015; World Health Organization, 2021). Phacoemulsification combined with intraocular lens (IOL) implantation is the only and effective surgical method for cataract. However, this therapy is rarely to handle the disease once and for all, as various long-term complications may occur after the IOL implantation. Posterior capsular opacification (PCO) contributes a significant portion in the postoperative complications after IOL implantation with high incidence, around 20–40% in adults and almost 100% in children (Awasthi et al., 2009; Karahan et al., 2014; Sen et al., 2019). It is proved that the PCO is mainly originated from the adhesion, proliferation, migration, and trans-differentiation of postoperative residual lens epithelial cells (LECs) (Werner, 2008; Jun et al., 2014). It will result in visual acuity decline and even blindness again. Although the PCO can be treated by the neodymium-doped yttrium aluminum garnet (Nd: YAG) laser capsulotomy, new complications including high intraocular pressure, macular edema, and even retinal detachment may occur after such surgery (Burq and Taqui, 2008; Karahan et al., 2014). Visual deterioration and loss arise from PCO not only worsen quality of patients’ life, but also aggravate the economic burden.

Numerous studies have been investigated to reduce the PCO incidence, such as surgical method improvement and new IOL-type design (Haripriya et al., 2017). In addition, there are more studies focused on IOL surface modification. Early investigations were focused on the hydrophilic modification on IOL surface, such as heparinization or pegylation, which decreased the initial cellular adhesion to inhibit PCO (Han et al., 2017; Huang et al., 2017; Han et al., 2018). The surface heparinized IOL has been commercialized, whereas the clinical investigations have demonstrated that there is no desired efficacy on PCO incidence in long term (Maedel et al., 2013). As a result, antiproliferative drugs were introduced into IOL modification to decrease the PCO incidence subsequently. The drug-eluting IOL design showed great potential in PCO prevention and inhibition, as the drug participation greatly decreased the cell proliferation and reduced the PCO effectively (Han et al., 2019; Huang et al., 2021; Qin et al., 2021). Nevertheless, the previous drug-eluting IOLs preparation is mostly complex, time-consuming, and high cost, such as layer-by-layer assembly and surface initiated free radical polymerization (Wu et al., 2015; Zhang et al., 2016; Wen et al., 2022). Therefore, they are not benefit for rapid industrialized production and clinical translation. By the foregoing reasons, the investigation of facile drug-eluting coating with rapid and economical preparation for IOL modification is urgently needed (Qie et al., 2022).

**SCHEME 1:**
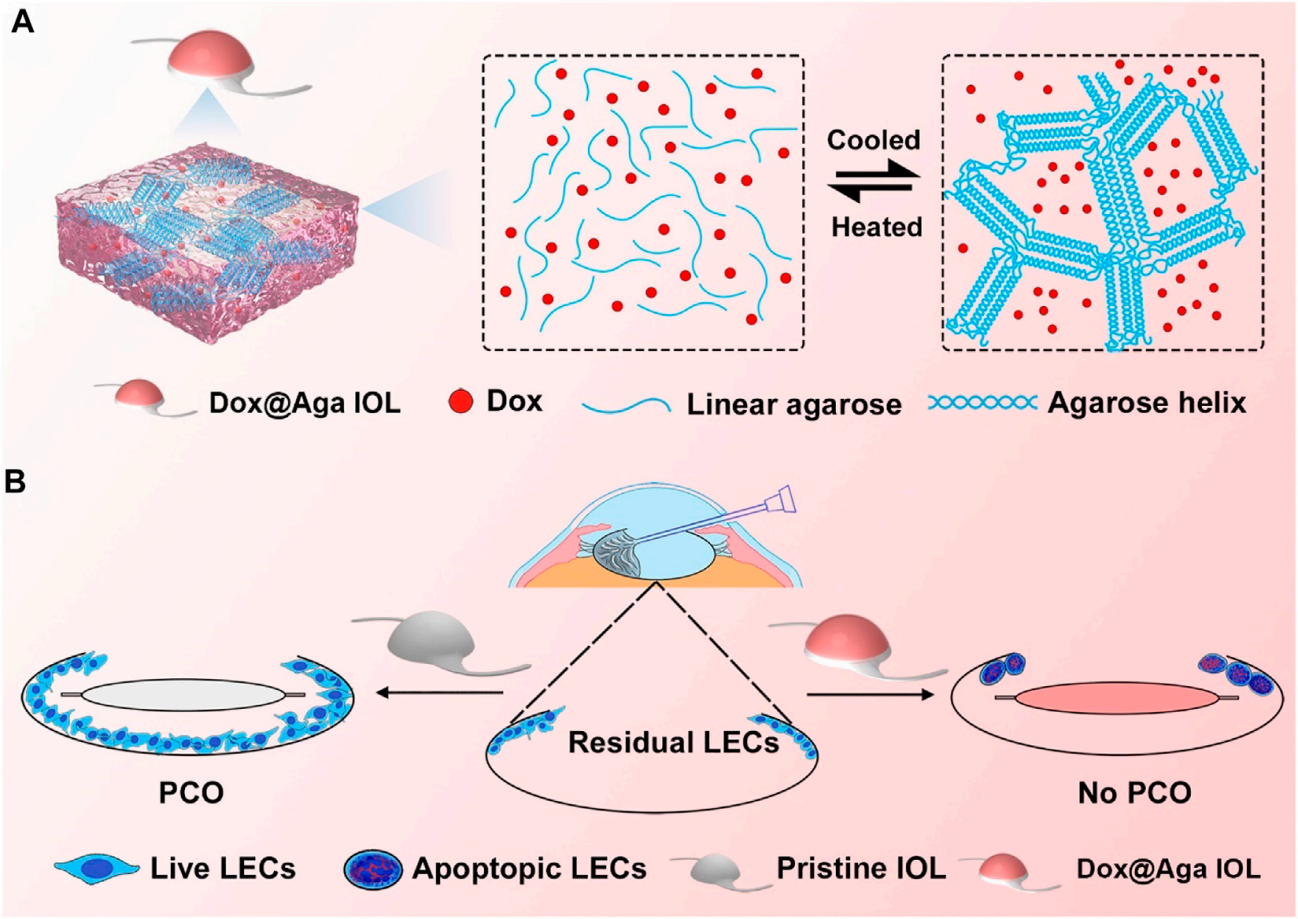
Schematic illustration of the gelation process of Dox@Aga coating **(A)** and effects of Dox@Aga coating on the PCO prevention after IOL implantation **(B)**.

Agarose (Aga) is a linear polysaccharide obtained from natural seaweed (Buckley et al., 2009). It consisted of alternately connected 1,3-linked *β*-d-galactose and 1,4-linked 3,6-lactone-l-galactose (Xu et al., 2005). Due to its nontoxicity, low immunogenicity, and outstanding biocompatibility, it has been widely used in biomedical application including tissue engineering, regenerative medicine, and more promising for drug delivery (Wu et al., 2017; Zarrintaj et al., 2019; Heo et al., 2020; Khodadadi Yazdi et al., 2020; Yang et al., 2020). In addition, Aga is a natural occurring thermoresponsive polymer with upper critical solution temperature (UCST) of 80–90°C. It is water soluble when the temperature is higher than 80°C. When the temperature decreases to lower than UCST, a lot of intramolecular and intermolecular hydrogen bonds as well as double helices form between the Aga chains, thus the gelation occurred (Russ et al., 2013; Zarrintaj et al., 2018). Moreover, it is reported that the Aga gel is sponge-like in macrocosmically, thus it is naturally a good drug depot (Ahuja and Pathak, 2009; Kim et al., 2019). Due to these specific features, the Aga may be used to fabricate drug-eluting coating on the IOL surface rapidly and economically. Herein, the drug-incorporated Aga gel coating via simple temperature alteration was investigated and introduced onto the IOL surface for PCO prevention purpose. The doxorubicin hydrochloride (Dox) was used as functional antiproliferative drug. As illustrated in Scheme1, Dox contained Aga solution was prepared under 90°C. The IOL materials were immersed in the coating solution for certain minutes and taken out into the room temperature. With the reduction of temperature, the Dox-incorporated Aga gel coating was obtained on the IOL surface rapidly and economically. The in vitro and in vivo PCO prevention effects of such drug-eluting IOL were also investigated.

## 2 Materials and Methods

### 2.1 Materials

Agarose (Aga, agarose with low electroendosmosis), calcein/PI live/dead cell assay kit, Hoechst 33342 staining solution, cell counting kit-8 (CCK-8), DiO cell plasma membrane staining kit, and hematoxylin/eosin staining kit were purchased from Beyotime Biotechnology Co. (Shanghai, China). Polyethylenimine (PEI, MW = 25,000) and fluorescein diacetate (FDA) were obtained from Sigma-Aldrich (St Louis, MO, United States). Doxorubicin hydrochloride (Dox) and advanced antifade mounting medium with DAPI were procured from Meilun Biotechnology Co., Ltd. (Dalian, China). Fetal bovine serum (FBS), 0.05% trypsin-ethylenediaminetetraacetic acid solution (Trypsin–EDTA), penicillin–streptomycin solution, Dulbecco’s modified Eagle’s medium/nutrient mixture F-12 (DMEM/F-12; 1:1 ratio), phosphate-buffered saline (PBS), and other cell culture-related reagents were purchased from Invitrogen (Waltham, MA, United States). Human lens epithelial cell line HLE B3 (CRL-11421™, LEC) was originated from American Type Culture Collection. Foldable hydrophobic IOL were obtained from 66 Vision Tech Co., Ltd. (Suzhou, China). The clinical postoperative administration drugs were supplied by the Eye Hospital of Wenzhou Medical University (Wenzhou, China). All other chemicals were of analytical grade and used without further purification.

### 2.2 Aga Coating Preparation

The medical polyester materials (polyethylene terephthalate), quartz, and commercialized IOL were used as the substrates for the Aga coating fabrication for adapting to different experimental and testing purposes. Briefly, the substrates were cleaned and immersed in 3 mg/ml PEI solution overnight so as to obtain the hydrophilic surface prior to Aga gel coating fabrication. The dip coating solution was prepared with a certain concentration of Aga and Dox in hot water (90°C). The pretreated substrates were soaked in coating solution at 90°C for a certain minutes and then taken out into the room temperature. Due to temperature changes, the Dox-loaded Aga coating (defined as Dox@Aga) was easily fabricated on the material surface. For optimizing the coating solution parameters, the coating obtained by different Aga concentrations (0 [pristine materials, without coating modification], 0.5, 1, 2, and 4 mg/ml Aga) and different Dox concentrations (0 [pure Aga coating, with no Dox added], 100, 200, 300, and 400 μg/ml) were prepared and investigated. To optimize the soaking time, 5, 15, 30, 60, and 120 min were used for investigation.

### 2.3 In Vitro Drug Release Assessment

In order to evaluate the Dox release behavior, the Dox@Aga substrates were released into a vial containing 3.3 ml PBS buffer solution (pH = 7.4). The 200 *μ*L Dox release solution was aspirated at predetermined time intervals (0.25, 0.5, 1, 2, 6, 12, 24, and 48 h, and 4, 7, and 14 days) from the vial and equal volume of fresh PBS were provided in the meanwhile. The experiment was carried out at 37°C to simulate intraocular physiological environment. Dox release solution was added into the photophobic well plates and then ascertained by the absorbance at 490 nm using a microplate reader. Thus, Dox content in release liquid can be calculated and curve of cumulative Dox release percentage could be drawn.

### 2.4 The Characterization of the Coating-Modified IOL Materials

The surface morphologies of the coating modified substrates were observed by the scanning electron microscope (SEM, Thermo Fisher Scientific, the Netherlands) and stereomicroscopy (SMZ1500, Nikon, Japan). The surface wettability was characterized by water contact angle analysis (WCA, OCA20, Data Physics Instrument GmbH, Germany). The light transmittance and absorbance of the Dox@Aga-coated substrates were examined by ultraviolet–visible spectrometer (UV–vis, 1780, Shimadzu, Japan). The drug loading density was calculated according to the absorbance of the coating eluent. The refractive index (RI) of the IOL with or without coating modification was performed by digital gem refractometer.

### 2.5 In Vitro Cell Behavior Evaluation of Aga Coating

#### 2.5.1 Cell Culture

The LEC were passaged and cultured according to the standard cell culture procedures (Lin et al., 2017). Briefly, the cells were cultured in DMEM/F-12 contained 10% FBS and 1% penicillin–streptomycin in an incubator at 37°C, 100% humidity, 95% air, and 5% CO_2_. After confluence, the cells were detached by 0.05% trypsin and resuspended in the culture medium and distributed onto cell culture plates containing the materials with a certain cell density.

#### 2.5.2 Biocompatibility Evaluation of the Aga Coating

For the biocompatibility evaluation, the sterilized leach liquors of the materials with or without Aga coating were prepared in advance. The cells were seeded in the 96-well plates at a density of 8 × 10^3^ cells per well. After 24 h of incubation, the cell culture medium was replaced by the 10% leach liquors supplemented cell culture medium and the cells were cultured for another 24, 48, and 72 h. The cells were stained by FDA and observed by the fluorescent microscope (DMi8, Leica, Germany). The cell densities on the wells in both groups were calculated and compared.

#### 2.5.3 Cell Elimination Effect Assay of the Dox@Aga Coating

For investigating the cell elimination effect of the Dox@Aga coating, the cells were seeded into the sterilized Dox@Aga coated materials layered on the 96 wells at density of 8 × 10^3^ cells per well. Serial Dox feeding concentrations, including 100, 200, 300, and 400 *μ*g/ml in the Dox@Aga coating preparation, were investigated. The pure Aga coating (Dox feeding concentration = 0 μg/ml) as well as the pristine material without coating modification served as controls. The cells were cultured for another 24, 48, and 72 h. The FDA staining and fluorescent microscopic observation were carried out at each time point. The cell morphology and distribution were also observed by SEM, calcein/PI staining, and Hoechst 33342 staining after another 24 h culture. The cell densities on different material surfaces were calculated and compared. The cell viability was also evaluated by the cell counting kit-8 (CCK-8) assay on a microplate reader (SpectraMax M5, Molecular Devices, United States) at an optical density of 450 nm.

The cells were also fluorescent co-stained by Hoechst 33342 and DiO to evaluate the cellular uptake of the Dox in the coating when cell cultured. In detail, as Dox had an autofluorescence, the cellular co-localization fluorescent images were taken using confocal laser scanning microscope, in which cell nuclei was stained by Hoechst 33342 and cell membrane stained with DiO. The DiO and Dox signals were simultaneously excited at 488 nm, while the emission wavelengths were 490–511 nm for DiO and 570–590 nm for Dox.

To monitor the cell migration and proliferation in lens capsule, the LECs were seeded at a density of 1.5 × 10^4^ cells per well in a circle along the periphery of the 24-well plates. After 24 h, the IOL materials with or without Dox@Aga coating modification as well as the pure Aga coating modification was placed into the center of each well. The cells were cultured for another 48 h and the antimigration effect was characterized by hematoxylin–eosin staining and stereomicroscopic images were taken under bright field.

### 2.6 In Vivo Animal Experiments

New Zealand White rabbits, 2.5 ± 0.5 kg in weight, were provided by the Wenzhou Medical University Animal Laboratory. The in vivo experiments were approved by the Laboratory Animal Ethics Committee of Wenzhou Medical University and all in vivo treatments were carried out in accordance with the Animal Experimentation Guidelines of the Wenzhou Medical University. The eyes were treated with levofloxacin eye drops before surgery to prevent infection and dilated with compound tropicamide eye drops. The involved surgical procedures for the intraocular IOL implantation were similar to that mentioned in our previous publication (Lin et al., 2017). In order to establish the PCO model, the polishing process was absent when doing the cataract surgery. The relevant examination of the eyes was also accomplished scrupulously before and after phacoemulsification combined with intraocular lens implantation. After the surgery, levofloxacin eye drops and tobramycin–dexamethasone ointments were applied to the operated eyes three times a day and once every night, respectively, during the first week to prevent postoperative inflammation. Compound tropicamide eye drops were also applied three times a day to prevent iris synechia. The acute ocular inflammation after surgery was observed by slit lamp microscope after 1 and 7 days. The intraocular pressure (IOP) of the rabbits in three groups was recorded with a contact tonometer (Tono-Pen, AVIA, United States). The anterior segment condition and PCO formation were obtained by slit lamp microscope postoperatively at 14 and 21 days. The specular microscope was used to observe the modality and density of cornea endothelial cells after the IOLs implantation. Then, these rabbits were euthanized and eyeballs were enucleated and immersed in 4% paraformaldehyde solution at 4°C. General photos and inspections of the Soemmering’s ring (SR) and PCO were also carried out to evaluate the development. The sections of the cornea, iris, retina, and the lens capsule were prepared and stained by hematoxylin–eosin for immunohistochemical analysis and viewed under the bright field microscope (DM4B, Leica, Germany). Opacification degree was graded from 0 to 4 (0 = none, 1 = minimal, 2 = mild, 3 = moderate, 4 = severe). The entire posterior capsule was divided into four parts and average scores of each part were calculated independently to evaluate the severity of PCO.

### 2.7 Statistical Analysis

At least three duplicates for each sample were set in the experiments. The results were expressed as the mean ± standard error of the mean. Statistical analysis, one-way analysis, and two-way analysis of variance were performed using GraphPad Prism (version 9.0). The significance level was set at *p* < 0.05. Differences with *p* > 0.05 were considered statistically insignificant, whereas those with *p* < 0.05 (*) were considered significant, and those with *p* < 0.01 (**), *p* < 0.001 (***), and *p* < 0.0001 (****) were considered very significant.

## 3 Results and Discussion

### 3.1 Optimization of Dip Coating Solution

**FIGURE 1:**
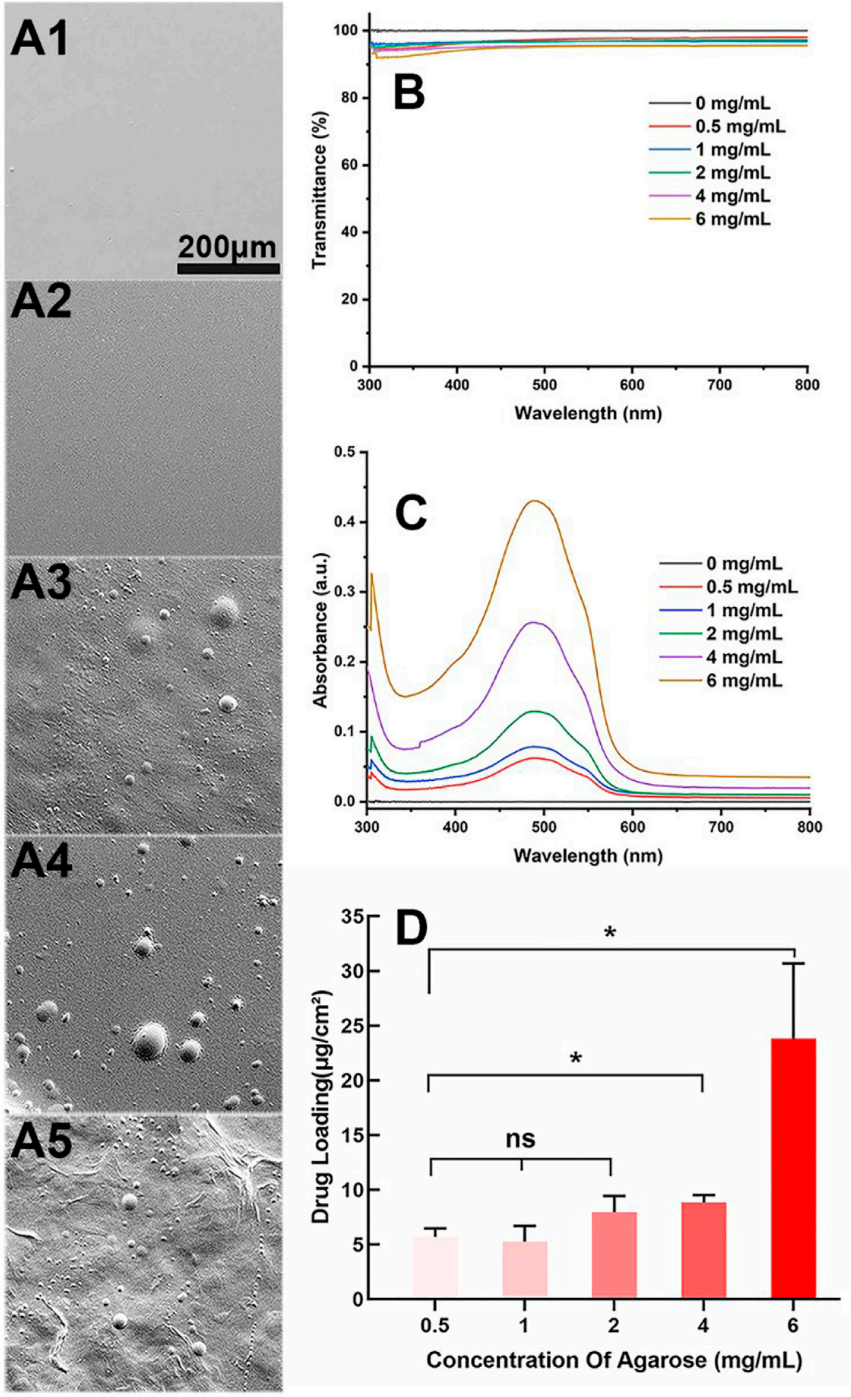
**(A)** Representative scanning electron microscope images of the Aga coatings obtained by different Aga concentrations **(A1–A5**: 0.5 mg/ml, 1 mg/ml, 2 mg/ml, 4 mg/ml, and 6 mg/ml, respectively, scale bar: 200 *μ*m**)**. **(B)** Visible light transmittance of the Aga coatings obtained from different Aga concentrations. **(C)** UV spectra of Dox-loaded Aga coating obtained from different Aga concentrations with same Dox concentration. **(D)** The calculated Dox loading density in the Aga coating obtained from different Aga concentrations. N = 6, ns means no statistical significance when compared each group with every other group at the level of *p* < 0.05 using ANOVA followed by a post hoc test.

Aga is a temperature-sensitive, thermoreversible, natural polysaccharide. The self-gelling behavior has been considered as its most fascinating feature, which is ascribed to the intramolecular and intermolecular hydrogen bonds formation when temperature decreases (Highley et al., 2019; Sun et al., 2019). Taking advantage of the self-gelling behavior, the Aga coating can be easily obtained on the material surface by simple dipping and temperature changing. Herein, various concentrations including 0.5 mg/ml, 1 mg/ml, 2 mg/ml, 4 mg/ml, and 6 mg/ml of Aga solution were prepared for coating preparation investigation. The surface morphology of each obtained coating was observed by SEM. As shown in Figure 1, the surface of Aga coating obtained from Aga concentrations of 0.5 mg/ml and 1 mg/ml were smooth and homogeneous (Figures 1A1, A2). The increasing of the Aga concentration results in more inhomogeneous layer on the coating surface. There are a lot of particle aggregations on the surface when the Aga concentration is higher than 2 mg/ml (Figures 1A3–A5). However, the light transmittances of these Aga coatings were all excellent. All of them were in the range of 94–96% (Figure 1B). As a refractive medium implant, the basic requisite is good light transmittance so as to meet the visual imaging acquirement. The excellent light transmittances of the Aga coatings indicate the great feasibility in the IOL coating applications. In this study, Dox was used as the model antiproliferative drug and added into the Aga solution. Thus, the drug-eluting IOL was obtained rapidly and economically, as the Dox can be loaded into the Aga coating when gelation on the surface. The ability of the Dox encapsulation into the coatings was investigated in the Aga solution with above different concentrations. As shown in Figure 1C, characteristic UV absorption peaks on the surface coating appeared when the Dox was added in the coating solution. Apparently the amount of loaded Dox increased with the Aga concentrations. However, there was no significant difference in the Dox loading densities as calculated from the coating eluents when the Aga concentration is lower than 2 mg/ml (Figure 1D), whereas the drug loading density was notably increased when the Aga concentration was higher than 2 mg/ml. It is proved that there was an exponential reduction in the pore size of Aga gel with the increasing concentration of the gelling Aga solution (Jokerst et al., 2011). As the Aga concentration increased from 0.5 mg/ml to 6 mg/ml, pore size of the Aga coating should be decreased dramatically. Thus, the inside network structure in Aga coating became more and more dense and hence drug molecules could be packed. Therefore, 0.5 mg/ml Aga was selected as the coating concentration for following investigations from the cost and environmental perspectives.

### 3.2 Optimization of Soaking Time

**FIGURE 2:**
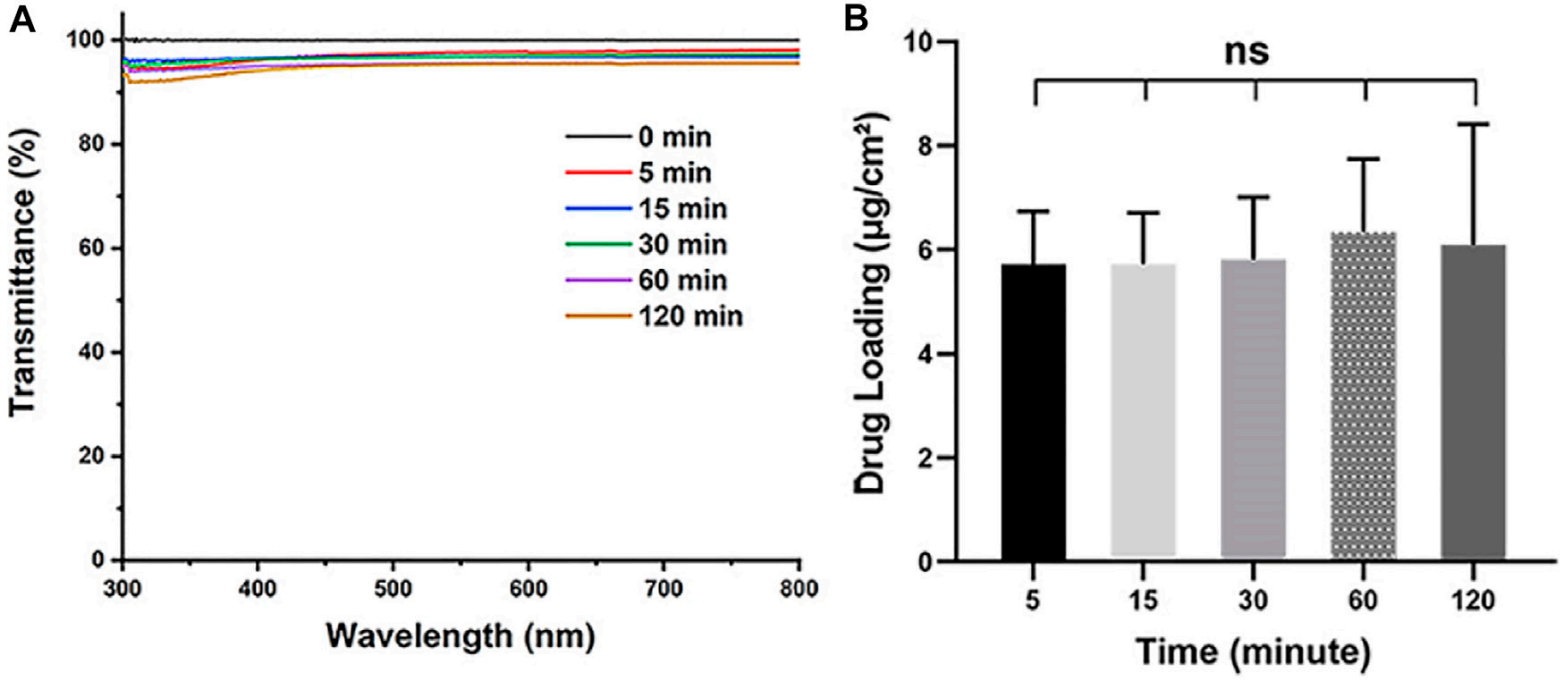
**(A)** Visible light transmittance of the Aga coatings obtained from different soaking time, including 0, 5, 15, 30, 60, and 120 min. **(B)** The calculated Dox loading density in the Aga coating obtained from different soaking time. N = 6, ns means no statistical significance when compared each group with every other group at the level of *p* < 0.05 using ANOVA followed by a post hoc test.

As aiming to realize the rapid preparation of IOL coating, the soaking time is also one of the important parameters when dip coating the Aga solution. The Aga coatings were prepared with optimized concentration (0.5 mg/ml) of Aga coating solution by different soaking time periods, from 5 to 120 min. The light transmittance and the drug loading density in the coating were also investigated. As shown in Figure 2A, the soaking time does not influence the light transmittance of the Aga coatings. The light transmittance of coatings obtained from various soaking time were in the range of 92–96% within the visible spectrum. There was no significant difference in drug loading densities among the Aga coatings obtained by different soaking time (Figure 2B), which indicated the drug loading amount in the coatings did not increase with the soaking time extension. Based on these results, 5 min was adopted as the soaking time due to the fast preparation purpose in the investigations.

### 3.3 Optimization of Dox Concentration Loaded in Coating

**FIGURE 3:**
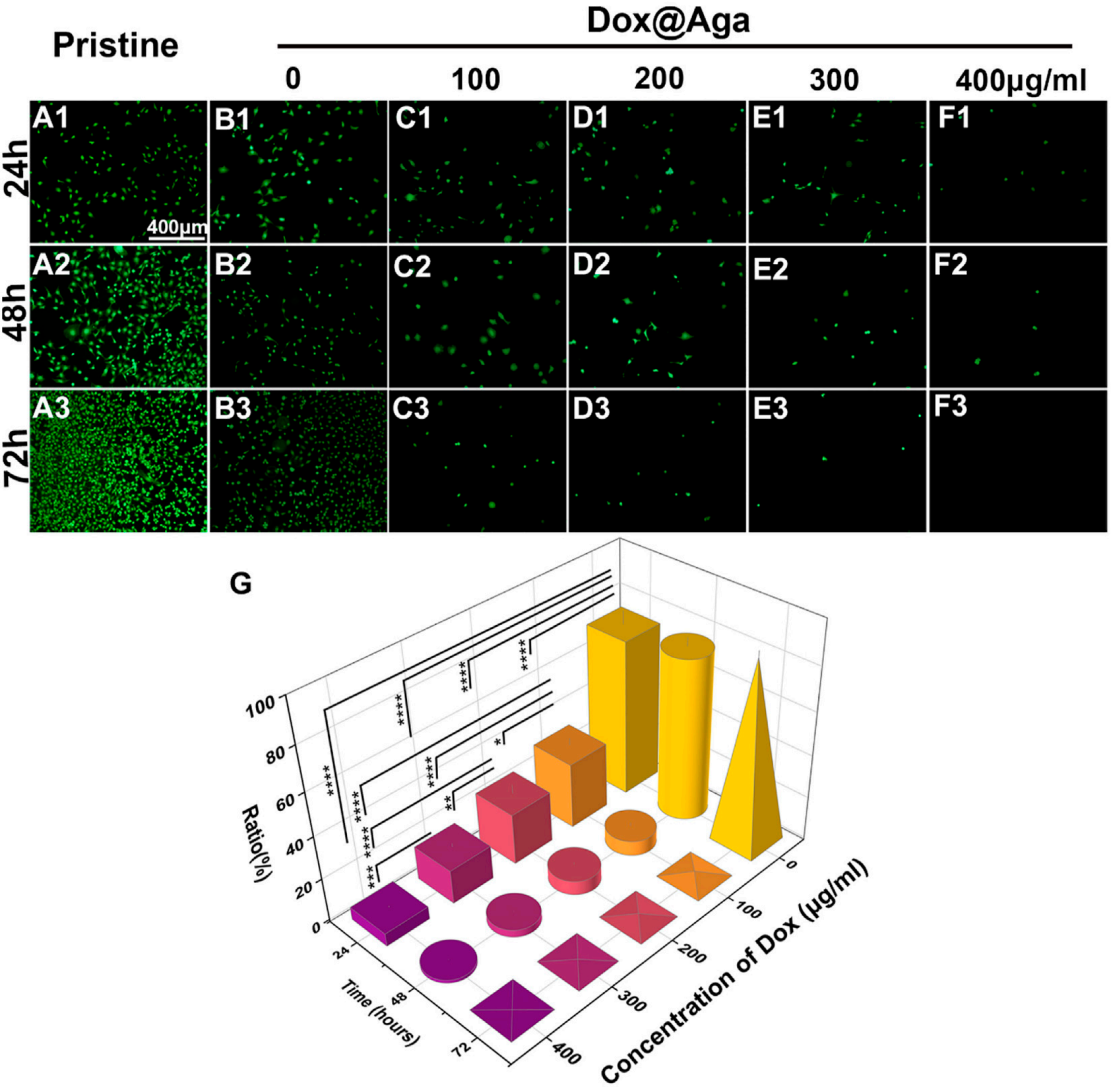
Representative fluorescent images of the FDA-stained LECs on the pristine and the different concentration of Dox-loaded Aga coating, cultured for 24 **(A1–F1)**, 48 **(A2–F2)**, and 72 h **(A3–F3)** (scale bar: 400 *μ*m). **(G)** The LECs survival rate ratio on the coating modified material surfaces. N = 5, ^∗^, ^∗∗^, ^∗∗∗^, and ^∗∗∗∗^ indicates *p* < 0.05.

After the parameter optimization of Aga concentration and soaking time, the optimization of the Dox concentration while preparating the coating solution was also conducted to ensure satisfied cytocidal effects. It is optimized by the cell elimination effect by in vitro cell culture. LECs were seeded on the Aga coating modified materials prepared by Aga solutions mixed with different concentrations of Dox (0, 100, 200, 300, and 400 mg/ml Dox in 0.5 mg/ml Aga solution, where 0 refer to the Aga coating without Dox). The obtained coatings were identified as Dox@Aga coating. The tissue culture polystyrene (TCPS) plates served as the control. FDA is an effective living cell dye for the cytoplasm staining. The observation of cell morphology and distribution were carried out by FDA staining and fluorescence microscope observation. The results were shown in Figure 3. It can be seen that the LECs grew in good growth state and proliferated vigorously on the unmodified pristine material surfaces (Figures 3A1–A3). The Aga coating modification moderately decreased the cell adhesion, as indicated by Figure 3B1, which may be due to the hydrogel nature of the Aga (Varoni et al., 2012; Seow et al., 2019). However, the cell proliferation is good (Figures 3B2, B3), which was in agreement with the good biocompatibility of the Aga. Moreover, when it comes to the Dox-loaded Aga coating, the cells amount decreased with the Dox concentration increasing and incubation time prolongation (Figures 3C1–F3). This is revealed intuitively by the 3D statistical map of the cellular survival rate on the Dox@Aga coating modified surface, comparing with the pristine materials (Figure 3G). Under the condition of 400 μg/ml Dox in the coating solution, the majority of LECs were cleared after 24 h, while the elimination went into the extreme after 72 h. As a result, the Dox concentration of 400 μg/ml was optimized and used in the coating solution.

As a result, taking the rapidity, economical efficiency, as well as effectiveness of IOLs coating purpose, the preparation conditions of the targeted Dox@Aga coating for IOLs surface modification were set as follows: coating solution was 0.5 mg/ml Aga with 400 mg/ml Dox, whereas coating time was 5 min. The Dox@Aga coating modified materials in the following investigations were prepared by such optimized parameters.

### 3.4 In Vitro Drug Release Assessment

The inside of Aga has a structure full of interconnected pores, which can ensure that nutrients, drugs, oxygen, and cells reach the surface of the Aga from the inside. In the study of Dox release behaviors, the Dox@Aga coating modified substrates were dipped in an environment similar to the physiological conditions in the eyes. As shown in Supplementary Figure S1, the release behavior of Dox was an outbreak releasing in the early stage, which reflected the release amount up to about 57% of the total. After which, an extended-release behavior was sustained and the release rate was slackened off. Based on the drug elution investigation, the loading amount of Dox in the such coatings was around 5.7 ± 0.9 μg and cumulative Dox release amount was up to about 57% of the total at 24 h, which is well below the upper bound cumulative dose for humans in clinical standard (Tan-Chiu et al., 2005). Moreover, some studies have shown that the proliferation of the LECs occurs mainly the first 4 days after the surgery (Saika et al., 2003; Gotoh et al., 2007). The Dox from Aga coating was released apace and in abundance, thereby residual LECs could be eliminated at an early stage, which is expected to effectively inhibit the development of PCO.

### 3.5 Characterization of the Aga Coating-Modified IOL

**FIGURE 4:**
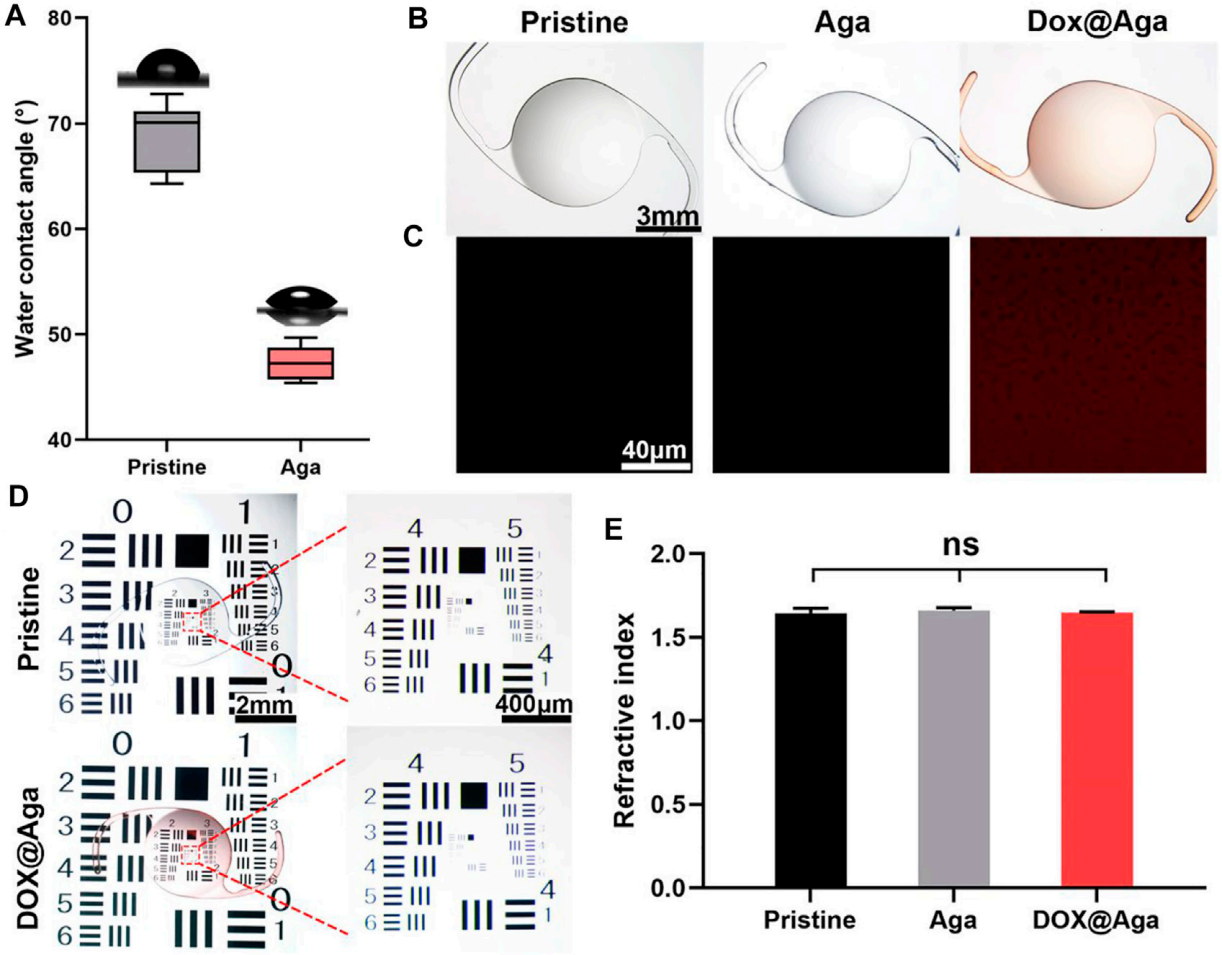
**(A)** Diagram summarizing water contact angle on different substrates. **(B)** The stereomicroscopic images of different IOL: the pristine IOL, the agarose gel coating IOL (Aga), and the agarose gel coating IOL loaded with Dox (Dox@Aga) (scale bar: 3 mm). **(C)** Confocal laser scanning microscopic images of the surface morphology of the corresponding substrates; the red fluorescence represented Dox (scale bar: 40 μm). **(D)** Imaging quality of the different IOLs characterized by the United States Air Force (USAF) resolution board (scale bar: 1 mm and 200 *μ*m). **(E)** The statistical diagram of refractive index of the three groups. N = 3, ns means no statistical significance when compared each group with every other group at the level of *p* < 0.05 using ANOVA followed by a post hoc test.

The surface wettability is one of the important properties of the IOL for their clinical applications. Currently, the most commonly used IOL types in the clinic are hydrophobic acrylic IOLs. With surface water contact angle (WCA) around 70–90°, silicone oil adhesion as well as lens epithelial cell adhesion and proliferation are prone to occur after their implantation. Surface hydrophilic modification proved to decrease these complications due to their antifouling properties (Lin et al., 2015; Han et al., 2017; Lin et al., 2017). Herein, the surface wettability was also investigated. As shown in Figure 4A, the Aga coating modification improves the surface hydrophilicity of the materials. The WCA changed from 74° to 46° after the Aga coating modification. The hydrophilic changes after Aga coating modification may be the origin of the foregoing cell adhesion decrease on such surface. The surface morphology was observed by stereomicroscope (Figure 4B). It can be observed that the Aga coating modification does not influence the surface topological morphology. The coating modified surfaces, no matter with or without Dox loading, were both homogenous and smooth. The apparently light red of the Dox@Aga coating also indicated the successful loading of the Dox. This was also confirmed by the confocal laser scanning microscopic observation (Figure 4C), as the autofluorescence of Dox was observed homogenously distributed in Dox-loaded Aga coatings. The imaging quality of the coating modified IOL was also investigated by the United States Air Force (USAF) resolution board (Figure 4D). According to the stereomicroscopic images, there was no obvious deviation or dimness after Dox@Aga coating modification when compared to the pristine IOL, which indicated that the Aga coating does not influence the imaging quality. The refractive index (RI) is another vital component of IOL optical performance. The RI testing results (Figure 4E) revealed that the RI values of the pristine IOL, Aga-coated IOL, or Dox@Aga-coated IOL were 1.64 ± 0.02, 1.66 ± 0.03, and 1.66 ± 0.01, respectively, which approved that the Aga coating modification does not have negative impact on optical properties of IOL.

### 3.6 Biocompatibility Evaluation of Agarose Gel Coating

**FIGURE 5:**
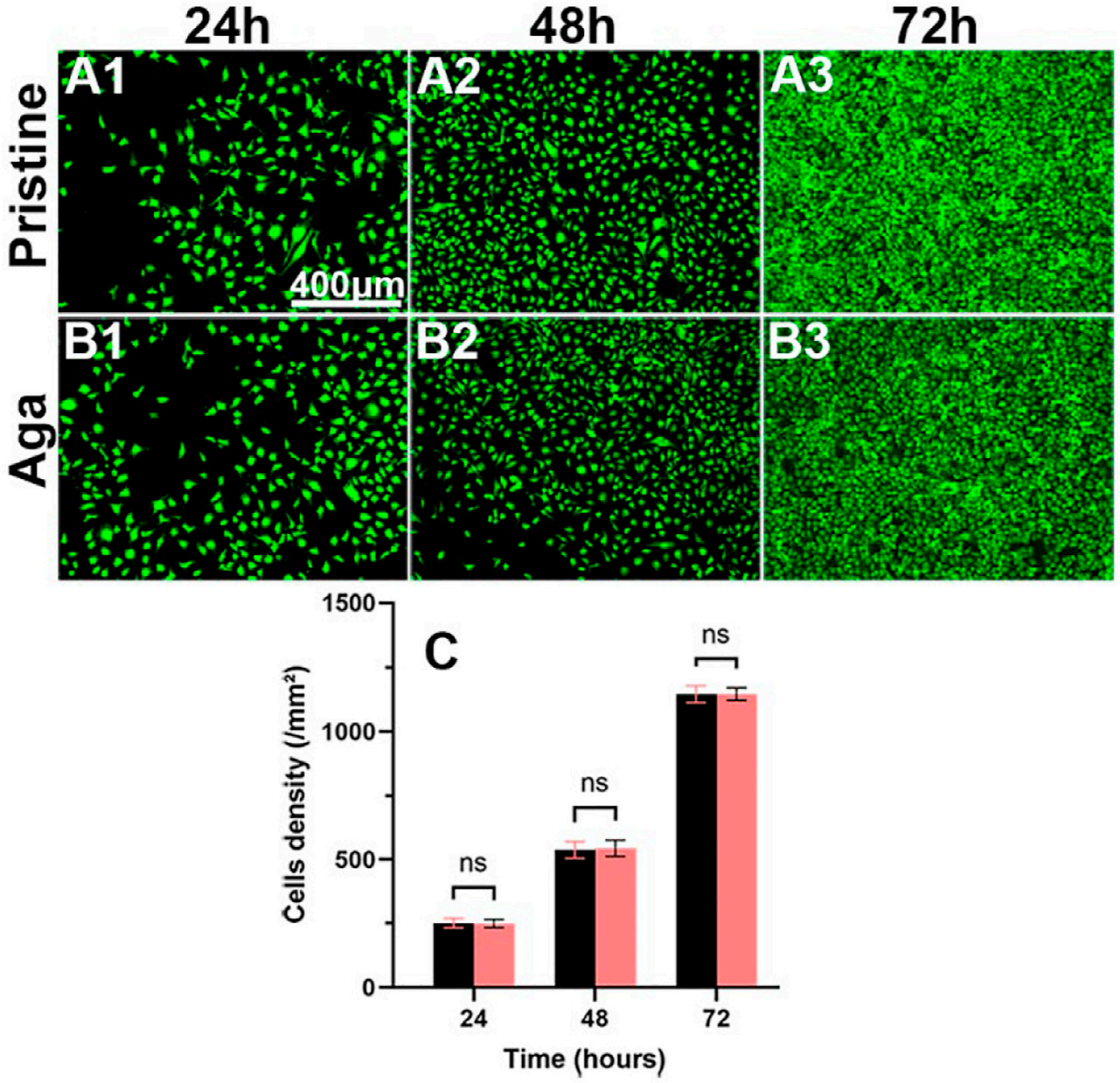
Representative fluorescent images of the LECs treated by the material leaching liquids with **(B1–B3)** or without **(A1–A3)** Aga coating modification, cultured for 24 h **(A1, B1)**, 48 h **(A2, B2),** and 72 h **(A3, B3)**. The green fluorescence represented FDA-stained LECs (scale bar: 400 *μ*m). **(C)** The calculated cell density. N = 6, ns means no statistical significance when compared each group with the other group for the same time point at the level of *p* < 0.05 using ANOVA followed by a post hoc test.

From the perspective of biosafety, implant materials are expected to be safe and nontoxic. The biocompatibility of Aga coating was evaluated through the cocultivation of leaching liquid and the LECs. Figure 5 shows the representative fluorescence images and density of the cells. The LECs treated with leaching liquid of the Aga coating modified materials grew well after 24 h (Figure 5B1), presenting a typical regular cellular morphology, as did it treated with the leaching liquid of the pristine materials (Figure 5A1). The cell morphologies after 48 h (Figures 5A2, B2) and 72 h cultures (Figures 5A3, B3) showed that the LECs of the two groups both proliferated rapidly. The mean cell density of these two groups was 250 ± 17 and 249 ± 15 for 24 h, and 537 ± 29 and 544 ± 29, 1,145 ± 31, and 1,146 ± 23 cells/mm^2^ after 24, 48, and 72 h culture, respectively. There was no significant difference found between the two groups (Figure 5C), and the results approve the excellent biocompatibility of the Aga coating, which is consistent with previous reports (Xu et al., 2016).

### 3.7 In Vitro Cell Elimination Assessment

The main purpose of the coating on the IOL was to eliminate the residual LECs for PCO prevention. Herein, the cell elimination effect of the Dox@Aga coating modified IOL materials were further assessed systematically.

#### 3.7.1 Antiproliferative Evaluation

**FIGURE 6:**
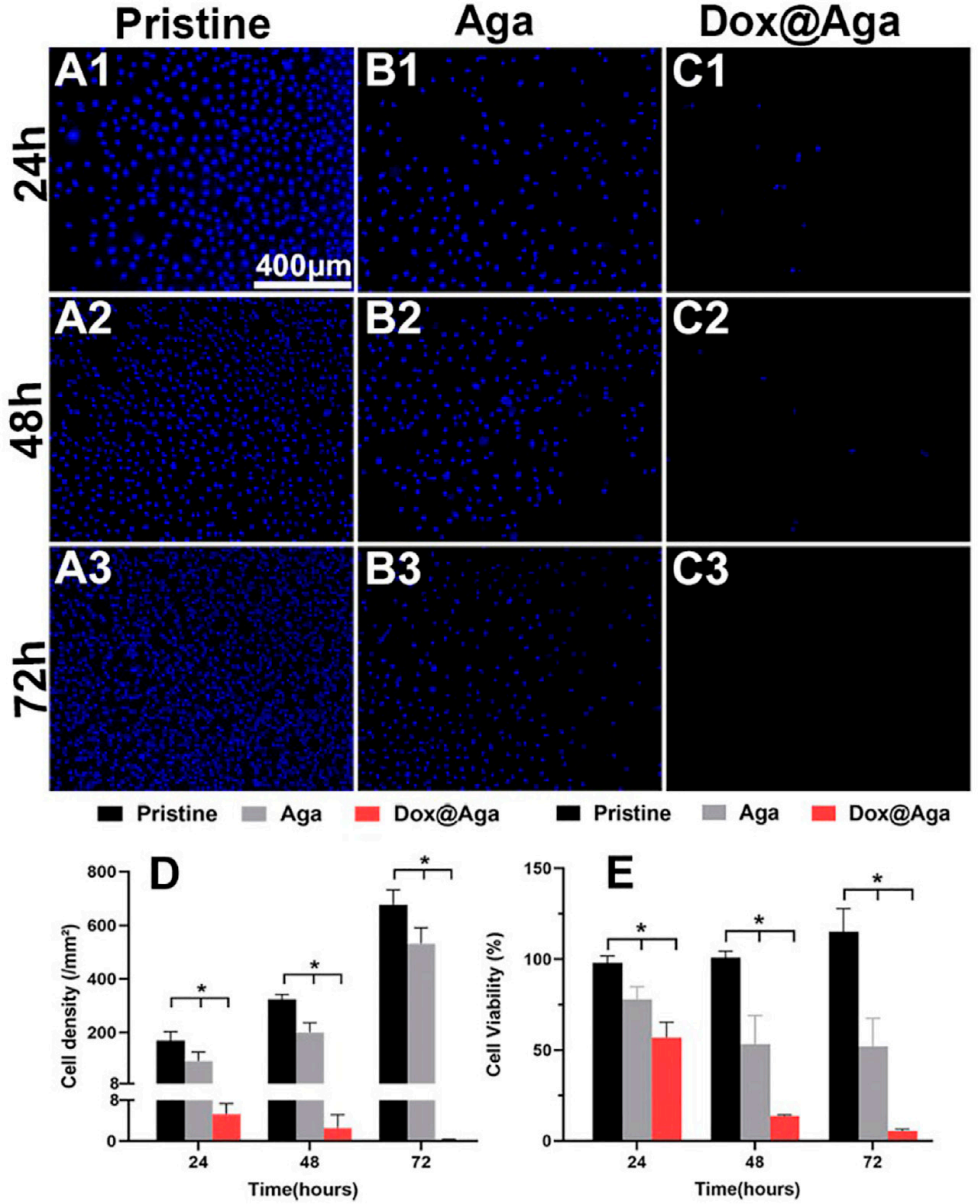
Representative fluorescent images of the Hoechst-stained cells on the pristine, the Aga coating modified, and the Dox@Aga coating modified materials surfaces with incubation time of 24 h **(A1–C1)**, 48 h **(A2–C2),** and 72 h **(A3–C3)**. The blue fluorescence represented cell nucleus (scale bar: 400 *μ*m). **(D)** The cell density of three groups, N = 5. **(E)** Statistical diagram of cell viability in three conditions. N = 6; ^∗∗^, ^∗∗∗^, and ^∗∗∗∗^ indicate *p* < 0.01.

The LECs proliferation is one of the key points in the earlier PCO development progress. In the Dox loading concentration optimization section, the cell was stained by the living cell cytoplasm staining dye FDA for exhibiting the cell abundance on the coating modified material surface. Herein, the LECs proliferation behavior was further confirmed by Hoechst staining and CCK-8 cell viability assays. The Hoechst is a dye that combines with cell nucleus and appeared blue under fluorescence microscopy. In consistence with the above cell investigation results, the cellular nucleus staining results indicated that the Aga coating decrease the cell attachment and the Dox@Aga coating could eliminate the cells on the surface effectively (Figures 6A–C). Seldom cells could be found on the Dox@Aga coating modified material surface when incubated for 24 h (Figure 6C1). And the cell proliferation was almost inhibited when incubated for 48 or 72 h (Figures 6C2, C3). The quantitation of the cell density on the material surfaces directly indicated the significant decrease of the cells on the surface (Figure 6D). Furthermore, the cell viability assay by CCK-8 also confirmed significant differences in cell elimination between the three groups (Figure 8E). All these results demonstrated that the Dox@Aga coating modification could inhibit the cell adhesion and proliferation effectively.

**FIGURE 7:**
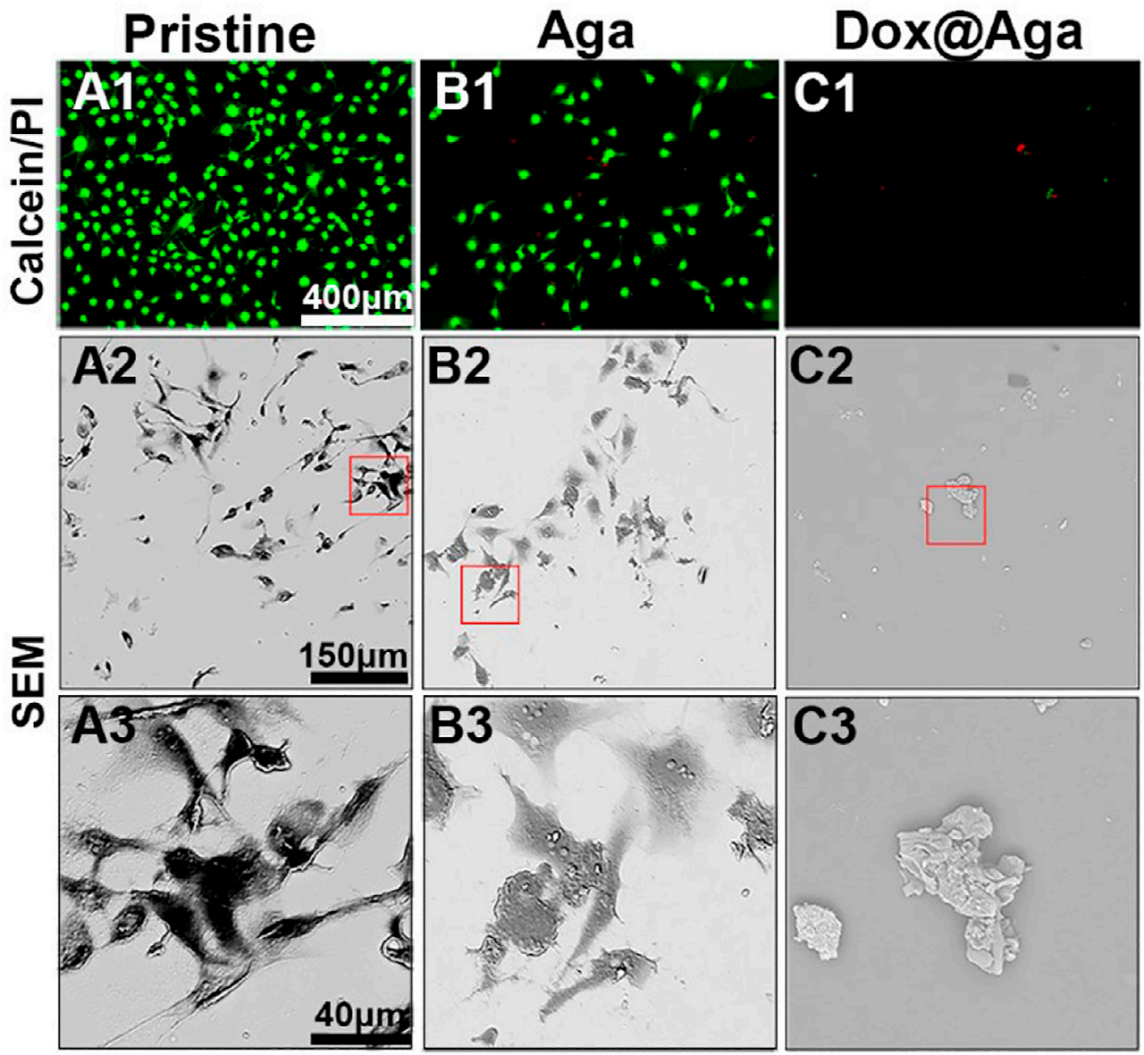
**(A1–C1)** Representative fluorescent images of the calcein/PI-stained cells, green and red fluorescence represented live and dead LECs, respectively. **(A2–C2, A3–C3)** The scanning electron microscope images of cell morphology on the three different kinds of material surfaces at different magnifications. **(A2–C2)**: ×500 and **(A3–C3)**: ×2000. Scale bar: 150 and 40 *μ*m, respectively.

The elimination effect was also confirmed by the live/dead (calcein/PI) staining assay of the cell on the material surfaces after cultured for 24 h. The cells with green fluorescence are alive, whereas with red fluorescence are apoptotic. As shown in Figures 7A1–C1, the antifouling effects of the Aga coating decrease the living cell adhesion. Meanwhile, the hydrophilic Aga coatings were undesirable for the cell proliferations, as some apoptotic cells were found on the Aga coating surface. As indicated in the results earlier, the Dox@Aga coating modification could effectively eliminate the cells on the material surface. Only few cells were found on the Dox@Aga coating modified material surface. Here, the few adherent cells were found to be apoptotic (Figure 7C1). These findings were also confirmed by the cell morphology observation by SEM (Figures 7A2–C2, A3–C3). LECs, seeded on the pristine IOL materials, exhibited abundant elongated and spindle-shaped pseudopodia (Figures 7A2, A3), which not only presents a good growth state, but also is the basis of cell migration. The cell spreading was less in the Aga coating surface, whereas LECs manifested emblematic apoptotic morphology with cell shrinkage, smaller size, and rounder shapes on the Dox@Aga coating surface.

#### 3.7.2 Dox Subcellular Localization

**FIGURE 8:**
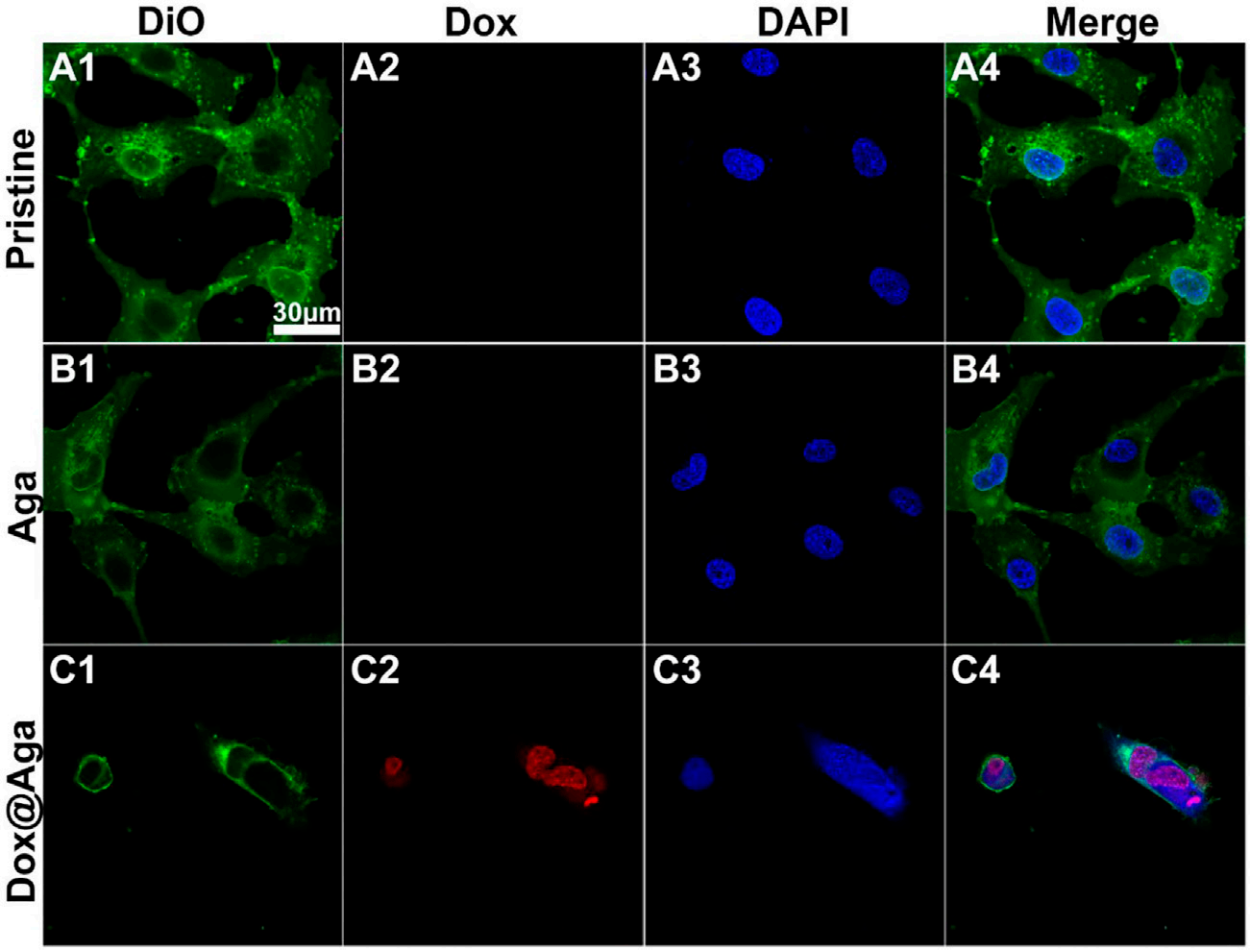
Representative fluorescent images of the LECs on the three different kinds of materials surfaces **(A4–C4)**. The cytomembrane **(A1–C1)**, Dox **(A2–C2)**, and nucleus **(A3–C3)** were represented by the green, red and blue fluorescence, respectively (scale bar:30 μm).

Dox triggers cell apoptosis by inserting into adjacent base pairs of DNA to generate active free radicals and break the DNA strand (Mizutani et al., 2005; Wei et al., 2020). Herein, the cell elimination mechanism was also briefly explored by the observation of Dox subcellular localization. Multi-fluorescence staining was carried out after cells were cultured on the material surfaces for 24 h and the confocal laser scanning microscopy was taken to present the subcellular localization of Dox in LECs. The Dox has red autofluorescence, and the nucleus and cytomembrane were stained by DAPI (blue) and DiO (green), respectively. As shown in Figure 8, the excellent cell spreading on the pristine materials and a bit less cell spreading on the Aga coating modified materials not only possessed dense cellular nuclei of compact form and high in number with round or oval and intact morphology, but also the cytomembrane displayed morphological integrity with extended and inter-contact pseudopodia. Nevertheless, the shrinkage of cytomembrane lead to an irregular outline of apoptotic cells, nucleus on the Dox@Aga coating surface exhibited typical apoptotic morphological feature, including obscure nucleus contours, nucleolar disintegration, and chromatin condensation. Red autofluorescence presented the cellular location of Dox, which demonstrated it entered indeed nucleus. This observation revealed the morphologies of normal growing and apoptotic LECs, which illuminated the effect of Dox on cells intuitively from subcellular localization level.

#### 3.7.3 Antimigration Evaluation

**FIGURE 9:**
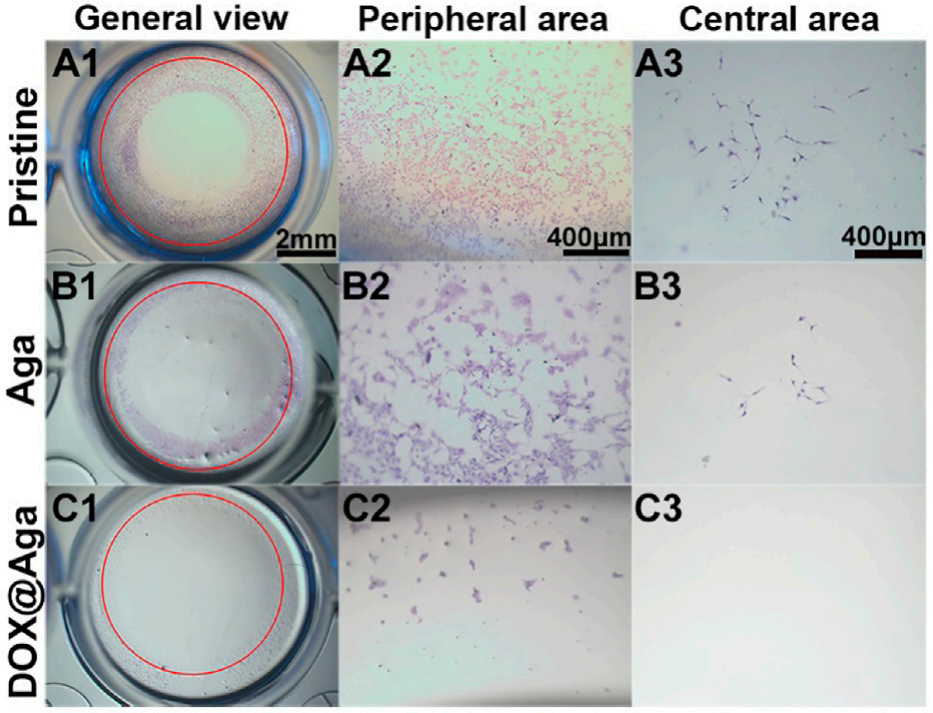
Cells seeding on three kinds of different materials surfaces in the 24-well cell culture plates to observe cell migration behavior. The representative photos taken by stereomicroscope from the general view **(A1–C1**; scale bar: 2 mm**)**, peripheral area **(A2–C2**; scale bar: 400 μm**)**, and central area **(A3–C3**; scale bar: 400 *μ*m**)**, respectively.

The above investigations demonstrated that the obtained Dox@Aga coating could eliminate the cell on its surface effectively. As the PCO is the result of residual LECs proliferation, migration toward central optical area of IOL in the lens capsular bag after cataract surgery proliferate and migrate in the capsular bag, the antimigration effect of such coating was also investigated by in vitro monitor. LECs were seeded along well plates and the coating modified materials were placed in the center of each well. The pristine material served as control. The antimigration was reflected in Figure 9. Red circles indicated the material edges. Significant cell migrations were found in the cases of pristine and Aga coating modified materials loaded wells. LECs proliferated beyond the material border and abundant cells migrated into the central area (Figures 9A1–A3, B1–B3). With regard to the Dox@Aga coating modified materials, not only cells migration was inhibited totally in its central area (Figure 9C2), but also characteristic apoptotic morphologies were observed in the peripheral area (Figure 9C3). These results indicated that the Dox@Aga coating could eliminate the LECs by antiadhesion and effectively inhibit the cell proliferation as well as migration, which should be a promising treatment for the prevention of PCO in vivo.

### 3.8 In Vivo Animal Experiments

**FIGURE 10:**
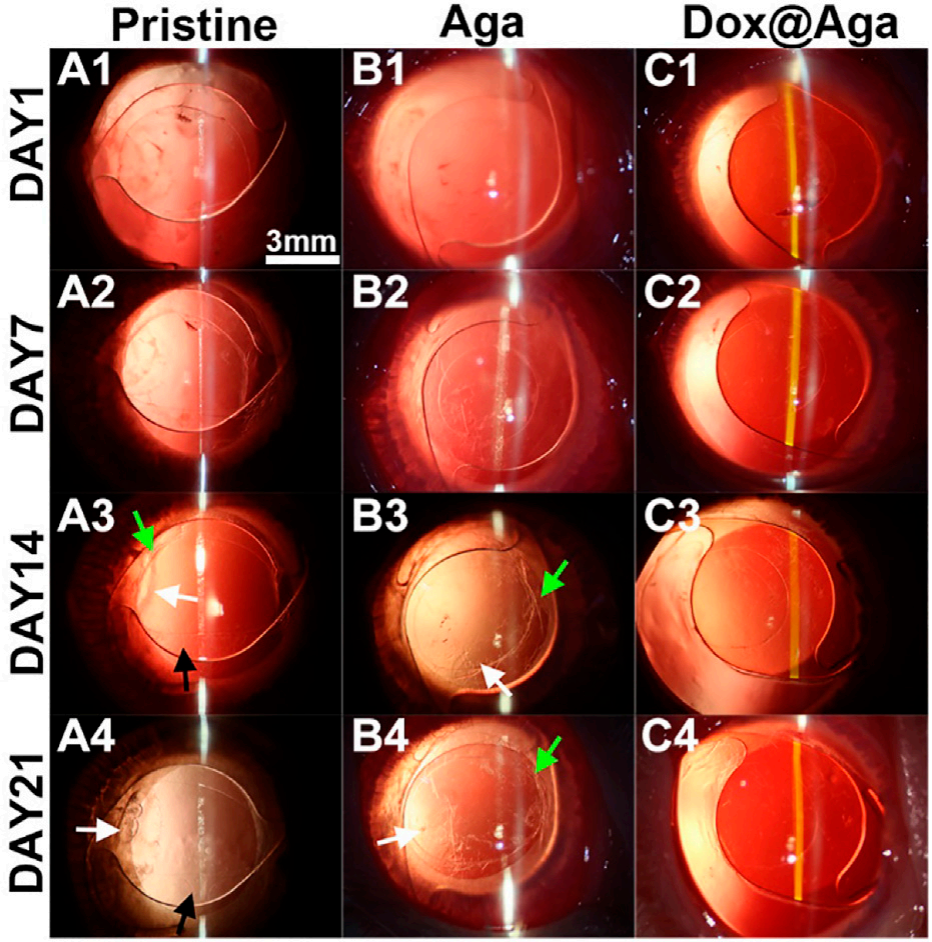
Representative slit lamp photographs of the rabbit eyes implanted with pristine IOLs **(A1–A4)**, Aga coating modified IOLs (Aga) **(B1–B4),** and Dox@Aga coating modified IOLs (Dox@Aga) **(C1–C4)**. The images were taken at postoperative days 1, 7, 14, and 21, respectively (black arrows indicated the scattered proliferating LECs; white arrows indicated the capsular wrinkles; scale bar: 3 mm).

**FIGURE 11:**
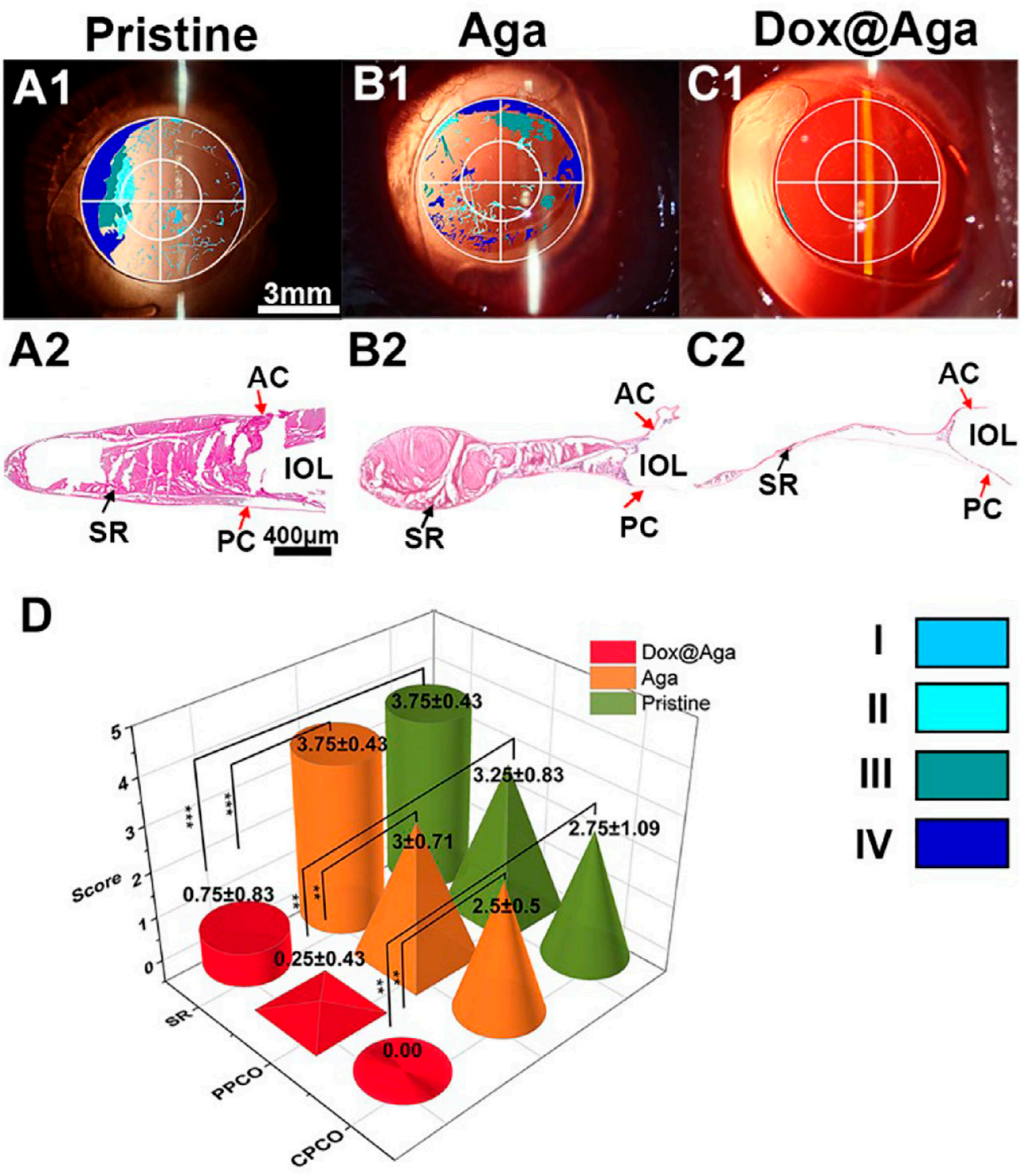
**(A1–C1)** The visualized images of PCO matched with slit lamp microscopic images (scale bar: 3 mm). **(A2–C2)** The H&E staining slices of the lens capsular bag of the eyes implanted with the pristine **(A)**, Aga coating modified **(B)**, and Dox@Aga coating modified **(C)** IOLs (scale bar: 400 μm). **(D)** PCO clinical evaluation and scoring analysis. Opacification density was graded from 0 to 4 (0 = none, 1 = minimal, 2 = mild, 3 = moderate, 4 = severe). ^∗∗^ and ^∗∗∗^ indicate *p* < 0.01.

The Dox@Aga coating modified IOLs were thus implanted into the animal eyes for evaluating the effect in PCO inhibition. The anterior segment changes and PCO developing progress were observed by the slit lamp microscope on 1, 7, 14, and 21 days postoperatively. Figures 10A1–A4, B1–B4, and C1–C4 were the representative images of the animal eyes implanted with pristine IOLs, Aga coating modified IOLs, and Dox@Aga coating modified IOLs, respectively. There were no acute inflammation and infection in all surgical eyes, and so no iris synechiae after surgery. Mild edema of cornea appeared in the pristine and Aga coating modified IOLs groups at day 1 postoperatively, which attributed to the surgery and receded under the treatment of anti-inflammatory eye drops. For the Dox@Aga-modified IOLs group, neither the edema appeared nor the exudation after surgery. Nevertheless, differences between each group emerged gradually at 2 weeks postoperatively and developed rapidly. Partial fibrosis and wrinkle of posterior capsule, as indicated by the white arrow, occurred in eyes implanted with pristine and Aga coating modified IOLs, 14 days after surgery, which were due to the pathological hyperplasia of residual LECs. In contrast, no posterior capsule shrinkage and fibrosis emerged in the eyes with Dox@Aga coating modified IOLs implantation in this period. It is noteworthy that scattered cells in abundant were observed at the posterior capsule in pristine IOLs group (black arrow in Figure 10A3) and did not appear in the Aga and Dox@Aga coating modified IOLs group eyes, which is in agreement with the results that the Aga coating surfaces repel the cell attachment. The hyperplasia deteriorated further and migrated toward the central optical region in pristine IOLs under observation for 21 days (Figure 10A4). Similar phenomena were found in the eyes with Aga coating modified IOLs, but it was less severe than that in the pristine IOLs (Figure 10B4). It revealed the limited PCO prevention effect of the only Aga coating modification, as it only delays but cannot inhibit the PCO incidence in the middle and long term. In contrast, the posterior capsule was fairly clear and transparent with no hyperplasia or obvious opacity in the eyes with Dox@Aga coating modified IOLs during the whole observation (Figure 10C4). To make the contrast most intuitive, combined with the evaluation of posterior capsule opacification image analysis (Tetz et al., 1997), areas that represented emblematic pathological changes of PCO and various degrees of opacification density of PCO fill with different colors (Figures 11A1–C1), which were subjectively divided into I, II, III, and IV, representing scattered cells (I), mild opacity (II), moderate opacity (III,) and severe opacity (IV). For the pristine IOLs group, white opacification had spread to the central optical region with the opacity in peripheral area of a wide range reaching level IV and abundant cells dispersed throughout the posterior capsule, which would be the basis of fibrous metaplasia. When it comes to the Aga coating modified IOLs group, there were no scattered cells but still with opacification invaded into central area. In contrast, posterior capsule had slight shrinkage with a high transparency in Dox@Aga group eyes.

After the whole observation period, rabbits were euthanized humanely and the eyeballs were enucleated and dissected to get lens capsule for the following assessments. Miyake-Apple view observations (Pereira et al., 2009) and histopathological section inspections of the lens capsule were also conducted to evaluate the preventive effect of coating modified IOL on PCO. As indicated in Figures 11A2, B2, a wide range of serious cortical hyperplasia existed in the IOL optical area, including heavy SR and severe anterior and posterior capsular hyperplasia (show in the red arrows of SR, AC, and PC, respectively). By comparison, these characteristic pathological changes were very slight in the Dox@Aga coating modified IOLs group (Figure 11C2), which confirmed the outstanding PCO inhibition effect of the Dox@Aga coating modification. Moreover, Miyake-Apple view observation was executed under stereomicroscope to analyze the degree and range of the white opacity more meticulously. In order to quantify the results to be more credible, the PCO scoring was analyzed in line with standard scoring criteria (Lin et al., 2015). The partition of PCO includes periphery PCO (PPCO) and central PCO (CPCO), which are on behalf of the periphery and central IOL optical areas, respectively. The central IOL optical part refers to the inner circle on IOL optical parts with a diameter of 3 mm, which is the area of the light penetration in the eye (Tang et al., 2021). As shown in Figure 11D, the scores of SR, PPCO, and CPCO are 3.75 ± 0.43, 3.25 ± 0.83, and 2.75 ± 1.09 in the pristine IOLs group, respectively. The SR, PPCO, and CPCO scores of Aga coating modified IOLs group were 3.75 ± 0.43, 3 ± 0.71, and 2.5 ± 0.5, respectively. They were slightly lower than those of the pristine IOLs group, whereas there was no significance. As for the Dox@Aga coating modified IOLs group, the scores of SR, PPCO, and CPCO were 0.75 ± 0.83, 0.25 ± 0.43, and 0.00 ± 0.00, respectively, which decreased sharply and had a significant difference with another two groups. All these results demonstrate that Dox@Aga coating modification can effectively inhibit the PCO development.

### 3.9 In Vivo Biocompatibility Evaluation

The safety and biocompatibility of Dox@Aga IOLs were either checked from the view of morphology, presented by the specular microscope, intraocular pressure (IOP) examination, and histological sections.

**FIGURE 12:**
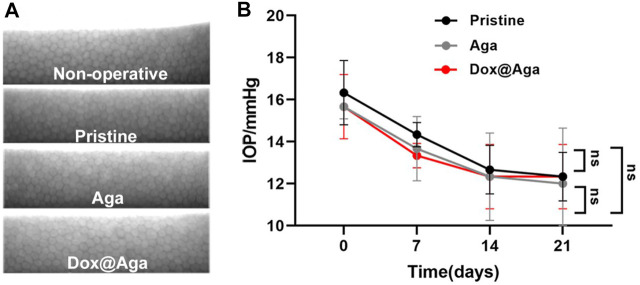
In vivo biocompatibility evaluation. **(A)** Representative specular microscopy images of corneal endothelial cells, including the nonoperative eyes and eyes implanted with the pristine, Aga coating modified, and Dox@Aga coating modified IOLs. **(B)** The intraocular pressure examination of eyes implanted with the pristine, Aga coating modified, and Dox@Aga coating modified IOLs (measurements were conducted at preoperative day, post-operative days 7, 14, and 21, respectively).

The specular microscope was used to visualize the corneal endothelial cells morphology and calculate the cell density. The operative eyes which were implanted with pristine IOLs, Aga IOLs, and Dox@Aga IOLs and nonoperative eyes were enrolled to compare. As shown in Figure 12A, the corneal endothelial cells exhibit a typical normal structure with regular hexagon at mosaic arrangement with clear boundaries and uniform density in each group. Besides, the statistical analysis of the IOP of all rabbits was shown in Figure 12B. After surgery, the IOP of all groups fell slightly but still remained within normal range. And there was no significant difference between each group.

**FIGURE 13:**
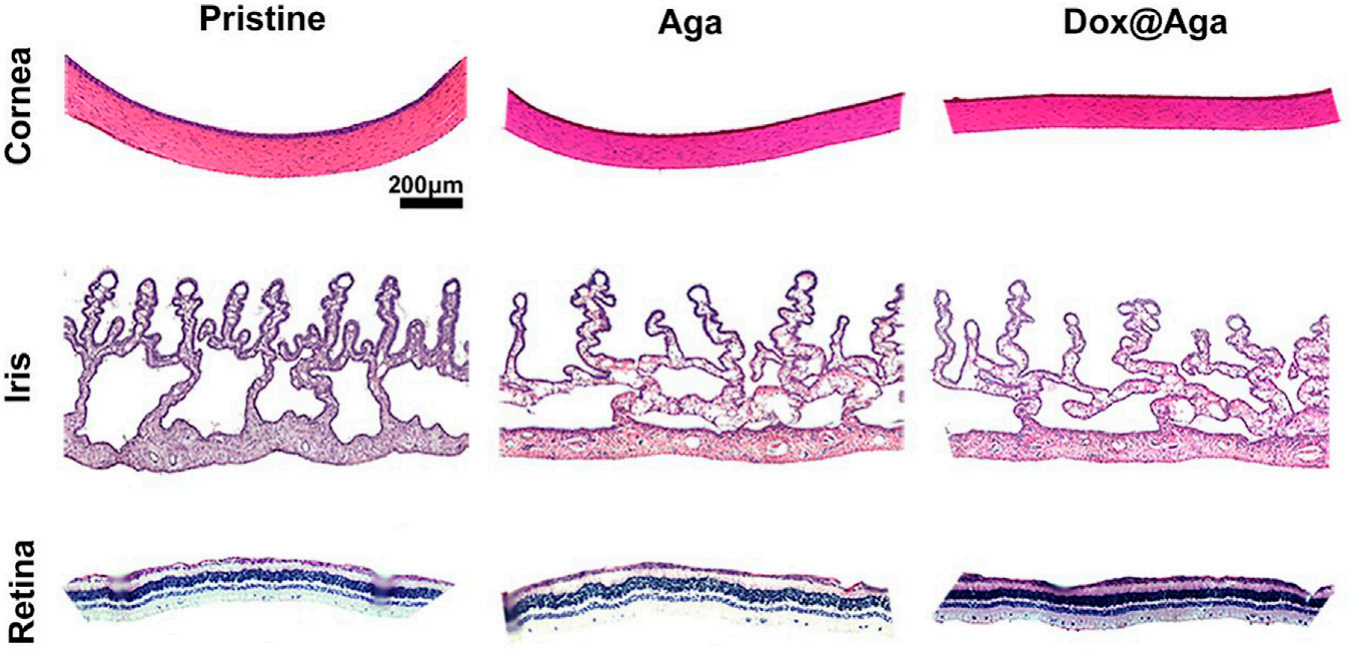
Representative H&E staining slices images of cornea, iris, and retina histomorphology after eyes implanted with the pristine, Aga coating modified, and Dox@Aga coating modified IOLs (scale bar: 200 *μ*m).

In addition to this, the cornea in the Dox@Aga group consisted of five-layer structure under bright field (Figure 13), which was in accordance with the morphology of normal cornea tissues. And the histomorphology of iris was normal with plentiful villi and blood vessels in Dox@Aga group, so did the pristine and Aga groups. The retina resides on the inner lining of the eyeball wall and plays a major role in eye imaging for its photoreceptive function. And there were no disorders presented in the retina structure. In general, all these results demonstrated the marvelous biocompatibility and safety of Dox@Aga IOLs.

## 4 Discussion

As the most common complication after cataract surgery, PCO is a confused clinical problem to be solved urgently. Modified IOL, the most widely studied in recent years, is the most promising means to realize clinical translation for PCO prevention. And more and more studies proved that drug-eluting IOL has more desirable effect on PCO prevention when compared with hydrophilic modified IOL.

However, PCO is closely related to the adhesion, proliferation, migration, epithelial mesenchymal transition (EMT), and fibrosis of LECs, extracellular matrix (ECM) deposition (Nibourg et al., 2015). Various signaling molecules would secrete during above process, all these substances that benefit cell proliferation and migration could accelerate PCO occurrence. For instance, adding the immunosuppressant cyclosporin A (CsA) into coating to modified IOL can effectively prevent PCO, also CsA induces autophagy-mediated cell death pathway in HLECs (Lu et al., 2022). In addition to this, the matrix metalloproteinases (MMPs) are a group of important endogenous proteolytic enzymes that can degrade extracellular matrix components, while the morphology, movement, and differentiation of the cells are all related to the ECM. Hydrophilic IOL with a MMP-2 enzyme response could reduce the severity of PCO (Han et al., 2020). In this research, the thermoreversible property of Aga was used and successfully realized rapid coating modification. Simultaneously, Aga was set as the drug delivery platform for its inner reticular structure to release Dox and eliminate residual LECs. Not only Dox, but also other drugs can be loaded in the coating to prevent corresponding disease, such as natural medicines, antimetabolites, anti-inflammatory agent, and immunosuppressant and molecules inhibitors. For example, antibacterial agents can also be loaded in Aga coating IOL to prevent endophthalmitis after cataract surgery. In summary, Aga coating drug-loaded IOL can be supported as a new treatment for PCO prevention, which is expected as the most promising option to realize clinical translation and commercialization.

When it comes to other applications of Aga, there are various fields. As a natural plant polysaccharide, there are many unique features of Aga which include repair of damaged tissues and organs. First, Aga has good biocompatibility and nontoxicity. Second, physicochemical property and feature of Aga is tunable. For example, Aga could be mixed with other materials to acquire the desirable mechanical properties. In addition, Aga has limited cell attachment, while it could increase cell affinity to agarose after chemical modifications. Third, the inside network structure of Aga gel is desirable platform for nutrients, oxygen, drugs, and even cells delivery. Based on aforementioned points, Aga and its derivatives and blends have been made into scaffold templates and widely used in tissue engineering, tissue regeneration, and drugs delivery, such as cartilage regeneration (García-Martínez et al., 2017) and mussel-inspired agarose hydrogel scaffolds for skin repair (Su et al., 2021).

## 5 Conclusion

In this study, a rapid and economical Aga coating was developed for drug-eluting IOLs fabrications on the purpose of PCO inhibition after intraocular implantation. Taking advantages of the temperature-sensitive property, Aga coating can be easily obtained by simple soaking in hot solution and room temperature cooling gelation process. The coating solution, including the concentrations of coating polymer (Aga) and the loading drug (Dox), was optimized on the purposes for the IOLs surface coating feasibility and LECs elimination effectiveness. The coating time was also optimized for the fast and economical fabrication objective. Such biocompatible Aga coating obtained at the optimized coating parameters does not influence the optical properties of IOLs, whereas it evidently improves the surface hydrophilicity, thus resulted in the cell adhesion repelling feature of such coating surfaces. The in vitro and in vivo investigations of the Dox@Aga coating modified IOLs demonstrated that such rapid and economical Dox-loaded Aga coating could eliminate residual LECs, and thus inhibit the PCO development effectively. Such drug-eluting IOLs by fast, convenient, feasible, and economical Aga coating technique with sufficient PCO prevention effect may have great potential in the clinical transformation.

## Data Availability

The original contributions presented in the study are included in the article/Supplementary Material; further inquiries can be directed to the corresponding authors.

## Supplementary Material

Supplementary Figure S1 Graph showing the release of Dox from Aga coating in PBS buffer (pH = 7.4). Dox release solution was aspirated at 0.25, 0.5, 1, 2, 6, 12, 24, and 48 h, and 4, 7, and 14 days. And all measurements were produced in triplicate and the mean concentrations and corresponding deviations were calculated.
