# Supplementary figures and images for: Rapid and Economical Drug-Eluting IOL Preparation via Thermoresponsive Agarose Coating for Effective Posterior Capsular Opacification Prevention
Source: Front Bioeng Biotechnol. 2022 Aug 5;10:930540. doi: 10.3389/fbioe.2022.930540 (PMC9388942; doi:10.3389/fbioe.2022.930540)

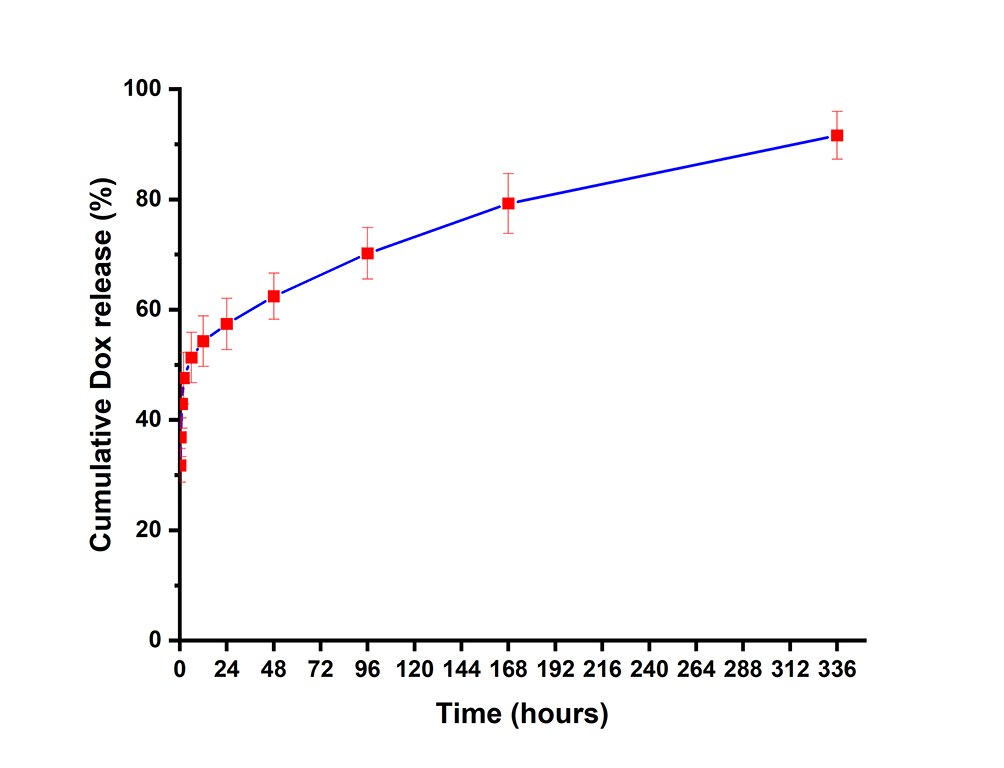

Supplement: Supplementary file 1 [file Image1.tif]
